# Fourier Approximation of magMEMS Oscillations: Neural Network Space Handling

**DOI:** 10.3390/mi16121355

**Published:** 2025-11-28

**Authors:** Piotr Skrzypacz, Arman Bolatov, Andrzej Dziedzic, Grant Ellis, Kaisar Tangirbergen, Bartosz Pruchnik, Piotr Putek

**Affiliations:** 1 Department of Mathematics, School of Sciences and Humanities, Nazarbayev University, Astana 010000, Kazakhstan; piotr.skrzypacz@nu.edu.kz (P.S.); arman.bolatov@nu.edu.kz (A.B.);; 2 Faculty of Electronics, Photonics and Microsystems, Wrocław University of Science and Technology, ul. Janiszewskiego 11/17, 50-372 Wrocław, Poland

**Keywords:** magMEMS, conservative singular oscillator, periodic solutions, dynamic pull-in, magMEMS switch, amplitude-frequency relation, neural networks, Fourier approximation

## Abstract

The initial value problem for a model of magnetic Micro-Electro-Mechanical System (magMEMS) with current-carrying conductors is considered. The model equation is a conservative MEMS oscillator due to the presence of the singular term corresponding to the magnetic force between two current-conducting wires. If the excitation parameter is below a certain threshold, the actuator oscillates. Fourier approximation of periodic solutions is enhanced by neural network space handling for both the limiting case where the geometric parameter approaches zero and the general case with arbitrary geometric parameter values. The method is validated through numerical comparisons with high-precision solutions, and its limitations are identified during the verification of experimental results. The findings can be useful for the design of magMEMS models.

## 1. Introduction

The operation of micro-electro-mechanical systems (MEMS) is strongly influenced by the pull-in effect, which occurs when electrostatic or magnetostatic forces overcome the restoring elastic forces, leading to mechanical instability and collapse. This phenomenon has been extensively investigated for electrostatically actuated MEMS devices [1,2,3]. The classical mass–spring model describing the pull-in effect was first introduced by Nathanson et al. [4], and further analyses clarified that the dynamic pull-in voltage is generally lower than the static pull-in threshold [5,6]. Although the electrostatic pull-in problem is now well understood, the corresponding analysis for magnetic MEMS (magMEMS) actuated by current-carrying conductors remains less developed. Understanding the oscillatory behavior and pull-in thresholds in magMEMS devices is crucial for ensuring reliable operation, particularly because excessive actuation currents can cause structural failure or permanent magnetic saturation [7,8,9,10,11]. Previous studies [12,13,14,15] have addressed dynamic pull-in and periodic oscillations for magnetic actuation models, emphasizing the need for accurate and computationally efficient approximation methods to guide device design.

Over the past two decades, various analytical and numerical techniques have been developed to approximate periodic solutions in nonlinear oscillatory systems. Among the most versatile methods is the Fourier–least squares method (FLSM) [16], which constructs periodic approximations by minimizing the residual of the governing nonlinear differential equation in the Fourier domain. This approach has been successfully applied to classical nonlinear oscillators, including the Duffing and Van der Pol systems [17], yielding accurate results even for strongly nonlinear regimes. Complementary to these classical methods, the rise of physics-informed neural networks (PINNs) [18] has opened a new avenue for integrating machine learning with differential equation modeling. Extensions of this framework, such as Fourier-feature-based PINNs [19], have demonstrated improved convergence for oscillatory solutions by embedding spectral information into the neural network input space. These developments motivate the combination of spectral and learning-based strategies for nonlinear problems with periodic responses.

The novelty of the present work lies in the formulation and application of a Fourier–neural network hybrid approach to compute approximate periodic solutions for magnetostatic MEMS oscillators with current-carrying wires. The proposed methodology combines the Fourier–least squares principle for residual minimization with neural network (NN) space handling, enabling efficient and high-accuracy computation of periodic orbits below the dynamic pull-in threshold. The approach is validated for both the limiting case of vanishing geometric parameter $\xi \to 0^{+}$ and for the general nonzero ξ configuration, providing quantitative insights into parameter dependencies and dynamic stability. This hybrid Fourier–neural framework enhances the analytical tractability and computational efficiency of nonlinear magMEMS modeling and can be extended to other classes of oscillatory systems with similar nonlinearities.

This paper is organized as follows. Section 2 introduces the mathematical model of the magMEMS device. In Section 3, we present the construction of approximate Fourier periodic solutions and the neural network-based determination of Fourier parameters. Section 4, in turn, extends the method to the general case and discusses validation and parameter dependencies, while Section 5 concludes the paper with remarks on the effectiveness and potential extensions of the proposed approach.

## 2. Mathematical Model of magMEMS Oscillator

**Figure 1:**
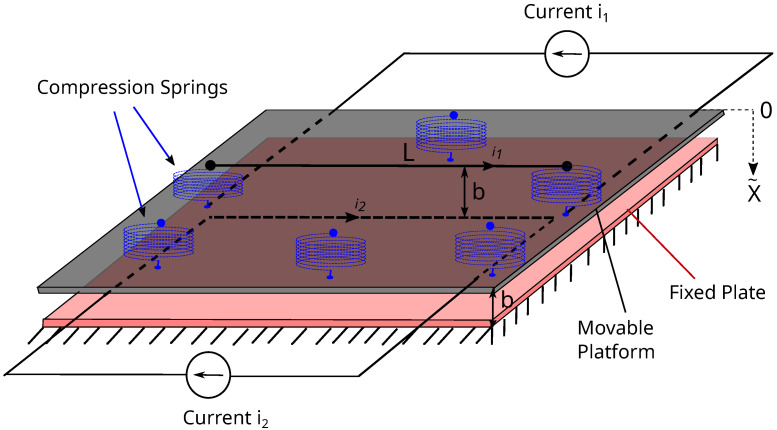
magMEMS with current-carrying filaments. The instantaneous distance between filaments is $b-\tilde{x}\left(t\right)$.

The magnetic force between two current carrying conductors having length *L* and separated by the distance *b* (Figure 1) can be expressed as [12]:

(1) $$F_{L}=\frac{i_{1}i_{2}\mu_{0}\mu_{r}}{2\pi }\left[\frac{b}{\sqrt{b^{2}+L^{2}}}+\frac{L^{2}}{b^{2}\sqrt{\frac{L^{2}}{b^{2}}+1}}-1\right]\;.$$

Then, the second Newton’s law of motion implies

(2) $$m\ddot{\tilde{x}}+k_{s}\tilde{x}-\frac{i_{1}i_{2}\mu_{0}\mu_{r}}{2\pi }\left[\frac{\left(b-\tilde{x}\right)}{\sqrt{{\left(b-\tilde{x}\right)}^{2}+L^{2}}}+\frac{L^{2}}{{\left(b-\tilde{x}\right)}^{2}\sqrt{\frac{L^{2}}{{\left(b-\tilde{x}\right)}^{2}}+1}}-1\right]=0,$$

where the restoring force due to the presence of a spring or springs with constant $k_{s}$ is

$$F_{R}=-k_{s}\tilde{x}\;,$$

and the attraction force between the current-carrying filaments due to the magnetic fields produced by the currents $i_{1}$, $i_{2}$ is given as

$$F_{L}=\frac{i_{1}i_{2}\mu_{0}\mu_{r}}{2\pi }\left[\frac{\left(b-\tilde{x}\right)}{\sqrt{{\left(b-\tilde{x}\right)}^{2}+L^{2}}}+\frac{L^{2}}{{\left(b-\tilde{x}\right)}^{2}\sqrt{\frac{L^{2}}{{\left(b-\tilde{x}\right)}^{2}}+1}}-1\right]\;.$$

The governing differential Equation (2) does not include a damping term with $\dot{\tilde{x}}$ since we assume that the air resistance and the mechanical damping of the flexible platform are negligible. Let us introduce the dimensionless distance and time as follows

(3) $$x=\frac{\tilde{x}}{b}\;\mathrm{and}\;t=\tilde{t}\sqrt{\frac{k_{s}}{m}}\;,$$

respectively. The dimensionless model equation then becomes

(4) $$\ddot{x}+x-K\left[\frac{\xi^{2}\;\left(1-x\right)}{\sqrt{\xi^{2}\;{\left(1-x\right)}^{2}+1}}+\frac{1}{\left(1-x\right)\sqrt{\xi^{2}\;{\left(1-x\right)}^{2}+1}}-\xi \right]=0\;,$$

where the scaled excitation parameter *K* is given by

(5) $$K=\frac{\mu_{0}\mu_{r}i_{1}i_{2}L}{2\pi \;k_{s}\;b^{2}}\;,$$

and

$$\xi =\frac{b}{L}$$

denotes the scaled geometric parameter. We complement Equation (2) with zero initial conditions

$$x\left(0\right)=\dot{x}\left(0\right)=0\;,$$

and assume that the currents in both wires are in the same direction or unidirectional, i.e., K≥0.

Multiplying both sides of Equation (4) by $\dot{x}$ and then integrating with respect to *t* leads to the conservation of the total energy

(6) $$\begin{array}{cc} E_{K,\xi }\left(t\right) & =\frac{1}{2}{\left(\dot{x}\left(t\right)\right)}^{2}+\frac{1}{2}x^{2}\left(t\right)\\ \; & \;-K\left[-\sqrt{\xi^{2}\;{\left(1-x\left(t\right)\right)}^{2}+1}+\mathrm{artanh}\left(\frac{1}{\sqrt{\xi^{2}\;{\left(1-x\left(t\right)\right)}^{2}+1}}\right)-\xi \;x\left(t\right)\right]\\ \; & \;-K\left[\sqrt{1+\xi^{2}}-\mathrm{artanh}\left(\frac{1}{\sqrt{1+\xi^{2}}}\right)\right]\;, \end{array}$$

from which it follows that

(7) $$\begin{array}{cc} {\left(\dot{x}\left(t\right)\right)}^{2}= & -x^{2}\left(t\right)\\ \; & \;+2K\left[-\sqrt{\xi^{2}\;{\left(1-x\left(t\right)\right)}^{2}+1}+\mathrm{artanh}\left(\frac{1}{\sqrt{\xi^{2}\;{\left(1-x\left(t\right)\right)}^{2}+1}}\right)-\xi \;x\left(t\right)\right]\\ \; & \;+2K\left[\sqrt{1+\xi^{2}}-\mathrm{artanh}\left(\frac{1}{\sqrt{1+\xi^{2}}}\right)\right]\;. \end{array}$$

Let

(8) $$\begin{array}{cc} f_{K,\xi }\left(s\right) & =-s^{2}+2K\left[-\sqrt{\xi^{2}\;{\left(1-s\right)}^{2}+1}+\mathrm{artanh}\left(\frac{1}{\sqrt{\xi^{2}\;{\left(1-s\right)}^{2}+1}}\right)-\xi \;s\right]\\ \; & \;+2K\left[\sqrt{1+\xi^{2}}-\mathrm{artanh}\left(\frac{1}{\sqrt{1+\xi^{2}}}\right)\right] \end{array}$$

be related to the righ-hand-side of Equation (7). The solution x(t) is periodic if the corresponding phase portrait in the $\left(x,\dot{x}\right)$ forms a closed curve. This holds true when the function $f_{K,\xi }\left(s\right)$ has a root in the interval (0,1). In the critical case $K=K_{\xi }^{*}$, the function $f_{K,\xi }\left(s\right)$ has a double root at $s=A_{\xi }^{*}$. This condition is satisfied when

(9) $$f_{K_{\xi }^{*},\xi }\left(A_{\xi }^{*}\right)=0\;\mathrm{and}\;\frac{\partial f_{K_{\xi }^{*},\xi }}{\partial s}\left(A_{\xi }^{*}\right)=0\;,$$

which results in the transcendental equation for $A_{\xi }^{*}$:

(10) $$\begin{array}{cc} \; & \frac{A_{\xi }^{*}}{2}\left[\frac{\xi^{2}\;\left(1-A_{\xi }^{*}\right)}{\sqrt{\xi^{2}\;{\left(1-A_{\xi }^{*}\right)}^{2}+1}}+\frac{1}{\left(1-A_{\xi }^{*}\right)\sqrt{\xi^{2}\;{\left(1-A_{\xi }^{*}\right)}^{2}+1}}-\xi \right]\\ \; & =-\sqrt{\xi^{2}\;{\left(1-A_{\xi }^{*}\right)}^{2}+1}+\mathrm{artanh}\left(\frac{1}{\sqrt{\xi^{2}\;{\left(1-A_{\xi }^{*}\right)}^{2}+1}}\right)-\xi \;A_{\xi }^{*}\\ \; & \;+\sqrt{1+\xi^{2}}-\mathrm{artanh}\left(\frac{1}{\sqrt{1+\xi^{2}}}\right)\;. \end{array}$$

**Figure 2:**
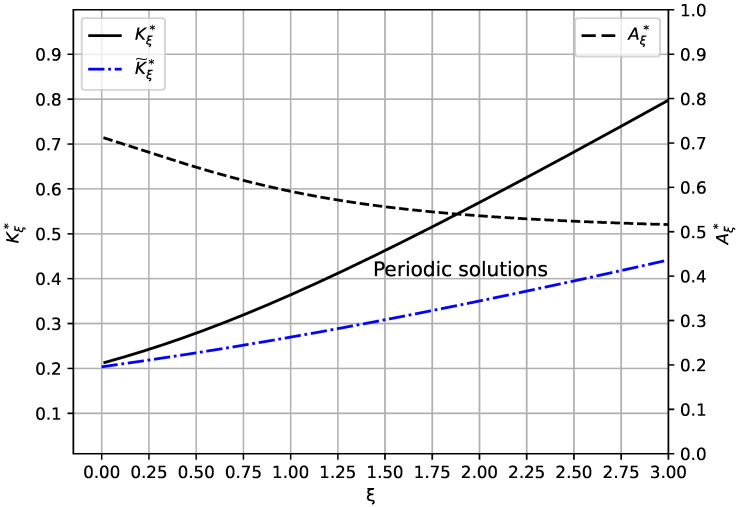
Maximum deflection $A_{\xi }^{*}$ of flexible part and dynamic pull-in threshold $K_{\xi }^{*}$ along with its lower bound ${\tilde{K}}_{\xi }^{*}$. Pairs (ξ,K) below the separatrix $K^{*}=K_{\xi }^{*}$ (solid line) lead to periodic solutions.

The value of $A_{\xi }^{*}$, representing the maximum deflection, can be obtained numerically for various values of the parameter ξ by solving Equation (10). Then, the corresponding dynamic pull-in threshold $K_{\xi }^{*}$ can be calculated as follows

(11) $$K_{\xi }^{*}={\left[\frac{\xi^{2}\;\left(1-A_{\xi }^{*}\right)}{\sqrt{\xi^{2}\;{\left(1-A_{\xi }^{*}\right)}^{2}+1}}+\frac{1}{\left(1-A_{\xi }^{*}\right)\sqrt{\xi^{2}\;{\left(1-A_{\xi }^{*}\right)}^{2}+1}}-\xi \right]}^{-1}A_{\xi }^{*}$$

due to the second condition from Equation (9). In Figure 2, the effect of the geometry parameter ξ on both the maximum deflection $A_{\xi }^{*}$ of the flexible platform and the dynamic pull-in threshold $K_{\xi }^{*}$ is illustrated. The maximum deflection $A_{\xi }^{*}$ decreases as ξ increases due to a weakening Lorentz force. For the same reason, the dynamic pull-in threshold $K_{\xi }^{*}$ increases with increasing ξ.

When the motion of the filament is caused by the magnetic field of an infinite current-carrying conductor, i.e., $\xi \to 0^{+}$, the zero initial value problem for Equation (4) becomes

(12) $$\begin{array}{cc} \ddot{x}+x-\frac{K}{1-x} & =0\;,\\ x\left(0\right)=\dot{x}\left(0\right) & =0\;. \end{array}$$

Multiplying (12) by $\dot{x}$ and integrating with respect to time, we get the conservation of energy

(13) $$E_{K,0}\left(t\right)=\frac{1}{2}{\left(\dot{x}\left(t\right)\right)}^{2}+\frac{1}{2}x^{2}\left(t\right)+K\ln\left(1-x\left(t\right)\right)\equiv 0$$

for all t≥0, and the corresponding function $f_{K,\xi }\left(s\right)$ defined in Equation (8) is given in the limit case ξ=0 by $f_{K,0}\left(s\right)=-s^{2}-2K\ln\left(1-s\right)$. The initial value problem by Equation (12) has periodic solutions if $K<K_{0}^{*}$, otherwise it has pull-in solutions. The dynamic pull-in threshold $K_{0}^{*}$ and the maximum deflection $A_{0}^{*}$ are given by [13,15]

(14) $$K_{0}^{*}=-\frac{2W_{-1}\;\left(-e^{-1/2}/2\right)+1}{4W_{-1}^{2}\;\left(-e^{-1/2}/2\right)}=0.2036321888\ldots \;,$$

and

(15) $$A_{0}^{*}=\frac{1+\sqrt{1-4K_{0}^{*}}}{2}=0.71533\ldots \;,$$

which can be deduced from Equation (9). In Equation (14), $W_{-1}:\left[-1/e,0\right)\to \left[-1,-\infty \right)$ denotes the second branch of the LambertW function which is defined as a solution y(x)≤−1 to the transcendental equation $ye^{y}=x$ for x∈[−1/e,0). The maximum deflection *A* satisfies for $0<K<K_{0}^{*}$ the transcendental equation

(16) $$A^{2}+2K\ln\left(1-A\right)=0\;.$$

The lower bound for the separatrix $K\left(\xi \right)=K_{\xi }^{*}$ can be inferred from Equation (11) as follows

(17) $$\begin{array}{cc} K_{\xi }^{*} & \geq \frac{\left(1-A_{0}^{*}\right)A_{0}^{*}\sqrt{\xi^{2}{\left(1-A_{0}^{*}\right)}^{2}+1}}{\xi^{2}{\left(1-A_{0}^{*}\right)}^{2}+1-\xi \left(1-A_{0}^{*}\right)\sqrt{\xi^{2}{\left(1-A_{0}^{*}\right)}^{2}+1}}\\ \; & =\left(1-A_{0}^{*}\right)A_{0}^{*}+{\left(1-A_{0}^{*}\right)}^{2}A_{0}^{*}\xi +\frac{\left(1-A_{0}^{*}\right)\left({\left(A_{0}^{*}\right)}^{2}-2A_{0}^{*}+1\right)A_{0}^{*}}{2}\xi^{2}+O\left(\xi^{4}\right) \end{array}$$

due to the fact that $A_{\xi }^{*}\geq A_{0}^{*}=0.715331863\ldots$, see Equation (15) and Figure 2. Notice that $K_{0}^{*}=\left(1-A_{0}^{*}\right)A_{0}^{*}$ according to the second condition in Equation (9). The lower bound ${\tilde{K}}_{\xi }^{*}$ from Equation (17) confirms that the dynamic pull-in threshold $K_{\xi }^{*}$ increases with increasing geometric parameter ξ as shown in Figure 2.

## 3. Approximate Periodic Solutions

### 3.1. Fourier Approximation

The differential equation in (12) can be rewritten as

(18) $$\left(1-x\right)\ddot{x}-x^{2}+x=K\;.$$

In order to obtain an approximate periodic solution of this conservative singular oscillator, we employ a Fourier approximation method. The key idea is to project the dynamics onto a subspace spanned by a finite number of basis functions that inherently satisfy the initial conditions. In our case, we consider the following ansatz:

(19) $$\hat{x}\left(t\right)=\alpha \sin^{2}\left(\omega t\right)+\beta \sin^{2}\left(2\omega t\right)+\gamma \sin^{2}\left(3\omega t\right)\;,$$

which guarantees that

$$\hat{x}\left(0\right)=0\;\mathrm{and}\;\dot{\hat{x}}\left(0\right)=0\;.$$

In Equation (19), the parameter ω denotes the frequency. The parameter vector with four parameters

θ=[ω,α,β,γ],

which include both a dominant frequency component and amplitude coefficients for the first three harmonics, are then determined by enforcing that the ansatz approximately satisfies the governing equation. After differentiating the ansatz and substituting into Equation (18), we obtain

(20) $$\begin{array}{cc} \; & 2\omega^{2}\left(\alpha \sin^{2}\left(\omega t\right)+\beta \sin^{2}\left(2\omega t\right)+\gamma \sin^{2}\left(3\omega t\right)-1\right)\\ \; & \;\times \left[2\alpha \sin^{2}\left(\omega t\right)-\alpha -32\beta \sin^{4}\left(\omega t\right)+32\beta \sin^{2}\left(\omega t\right)-4\beta +18\gamma \sin^{2}\left(3\omega t\right)-9\gamma \right]\\ \; & \;+\alpha \sin^{2}\left(\omega t\right)+\beta \sin^{2}\left(2\omega t\right)+\gamma \sin^{2}\left(3\omega t\right)\\ \; & \;-{\left(\alpha \sin^{2}\left(\omega t\right)+\beta \sin^{2}\left(2\omega t\right)+\gamma \sin^{2}\left(3\omega t\right)\right)}^{2}=K. \end{array}$$

Denoting the left-hand side of Equation (20) by $f_{\theta }\left(t\right)$, we define a loss function as the mean squared error

$$G\left(\theta \right)=\frac{1}{N}\sum_{i=1}^{N}{\left(f_{\theta }\left(t_{i}\right)-K\right)}^{2}\;,$$

where in our implementation, the residual $f_{\theta }\left(t_{i}\right)-K$ is computed at N=100 equidistant points $t_{i}$ in the time interval [0,5π]. This range was chosen because it covers several periods of $\hat{x}\left(t\right)$. Our goal is to minimize G(θ) over the parameter vector θ. This is achieved by an unconstrained gradient descent procedure.

### 3.2. Minimization Strategy

For each fixed value of *K*, the initial guess for the parameters was chosen as

θ=[1,K,K/2,K/4].

This choice is motivated by physical intuition: for small values of the excitation parameter *K*, the amplitude of the oscillations is expected to be small and to scale approximately linearly with *K*, while the dominant oscillatory behavior remains near the natural (or fundamental) frequency of the linear part of the system (here, approximately unity). Although simple stochastic gradient descent [20] or SGD with momentum can be used to update θ, our experiments showed that the Adam optimizer [21] converged significantly faster and more robustly. The Fourier residual is computed using PyTorch’s (PyTorch Stable (2.9.0) release) automatic differentiation [22], and the parameter vector is updated iteratively over 1000 iterations. Details of the optimization algorithm are as follows:

1. **Initialization:** For a given *K* (with $K<K^{*}$), initialize the parameter vector
  θ=[1,K,K/2,K/4]
  and generate *N* equidistant time points $t_{i}\in \left[0,5\pi \right]$.
2. **Loss Evaluation:** Compute the Fourier residual $f_{\theta }\left(t_{i}\right)$ at each $t_{i}$ and evaluate the mean squared error loss
  $$G\left(\theta \right)=\frac{1}{N}\sum_{i=1}^{N}{\left(f_{\theta }\left(t_{i}\right)-K\right)}^{2}.$$
3. **Gradient Update:** Use the Adam optimizer to update θ by backpropagating the gradient of G(θ).
4. **Iteration:** Perform the update for a fixed number of iterations (e.g., 1000 iterations). At each iteration, compare the current loss with the best loss recorded so far and save the corresponding θ if the current loss is lower.
5. **Output:** After the final iteration, output the parameter vector θ that achieved the minimum loss along with its corresponding loss value.

### 3.3. Comparison with High-Precision Runge-Kutta ODE Solver

**Figure 3:**
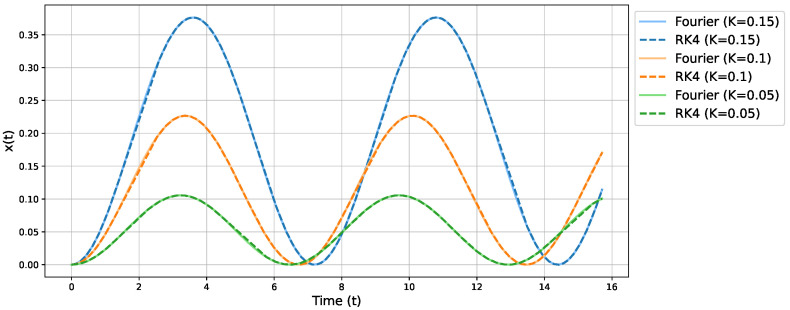
Comparison of the Fourier approximation (solid lines) with a high-precision Runge-Kutta solution (dashed lines) for three different values of *K*.

We validate each approximate solution by comparing it to a high-precision Runge-Kutta (RK4) integration of

$$\left(1-x\right)\;\ddot{x}\;-\;x^{2}\;+\;x\;=\;K,$$

with Δt chosen as $10^{-6}$ and with a precision of $10^{-8}$. Figure 3 illustrates these comparisons for three representative values of *K*. The solid lines show the Fourier-based approximations, whereas the dashed lines represent the RK4 solution sampled at several time steps. We observe excellent agreement.

**Figure 4:**
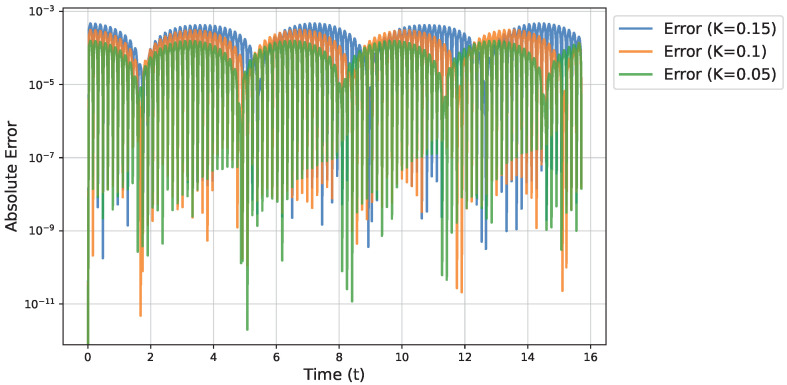
Absolute error between the Fourier-based approximation and the Runge-Kutta solution for different values of *K*. Note the logarithmic scale on the vertical axis.

To further quantify the accuracy, we compute the absolute error between the Fourier approximation and the RK4 solution, sampled on the same temporal grid, and plot this in Figure 4. We find that an error remains small for ω and α, with slightly larger deviations on β,γ. The Fourier-based method captures the behavior of the larger modes, but it underestimates the amplitudes of the highest-frequency terms somewhat.

### 3.4. Neural Network Fitting of Amplitude-Frequency Parameters

Although we can solve for θ=[ω,α,β,γ] repeatedly for each individual *K* black by minimizing L(θ), it is often convenient to have an explicit (or near-explicit) mapping from *K* to the parameters [ω,α,β,γ]. We accomplish this by training a single neural network whose output layer has four nodes, corresponding to ω(K), α(K), β(K), and γ(K). Concretely, we sample *K* at various points in $\left(0,\tilde{K}\right]$, with $\tilde{K}$ near the dynamic pull-in threshold $K^{*}$, and for each *K* we find the best-fit θ that minimizes L(θ). This yields a training set $\left\{\;\left[K_{i},\;\omega_{i},\;\alpha_{i},\;\beta_{i},\;\gamma_{i}\right]\;\right\}$.

The neural network is a fully connected feedforward 4-layer net with widths of 64, 128, 64, and 32 nodes, using GELU [23] activations for smoothness. Specifically, this activation is defined as

$$\mathrm{GELU}\left(x\right)=\frac{x}{2}\left[1+\mathrm{erf}\left(\frac{x}{\sqrt{2}}\right)\right],$$

where erf denotes the error function. We optimize for up to 500,000 epochs, employing the Adam optimizer and a cosine-annealing learning rate schedule [24]. An early stopping criterion is used based on a patience threshold to halt training once no further improvement in the loss is observed.

**Figure 5:**
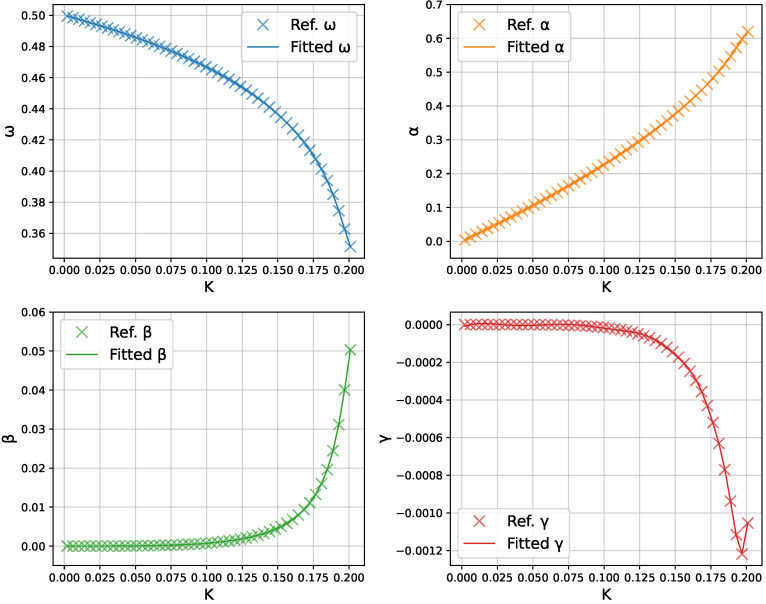
Neural-network fitting of the parameters ω,α,β,γ as functions of *K*. Crosses (×) represent the individual solutions obtained from the Fourier-residual minimization; solid lines show the neural network predictions.

Figure 5 shows a direct comparison of the neural-network-predicted profiles of ω(K),α(K),β(K), and γ(K) in the range of 0≤K≤0.2 versus the true values obtained from repeated least-squares fits of θ. The network captures the parameter trends very accurately.

**Figure 6:**
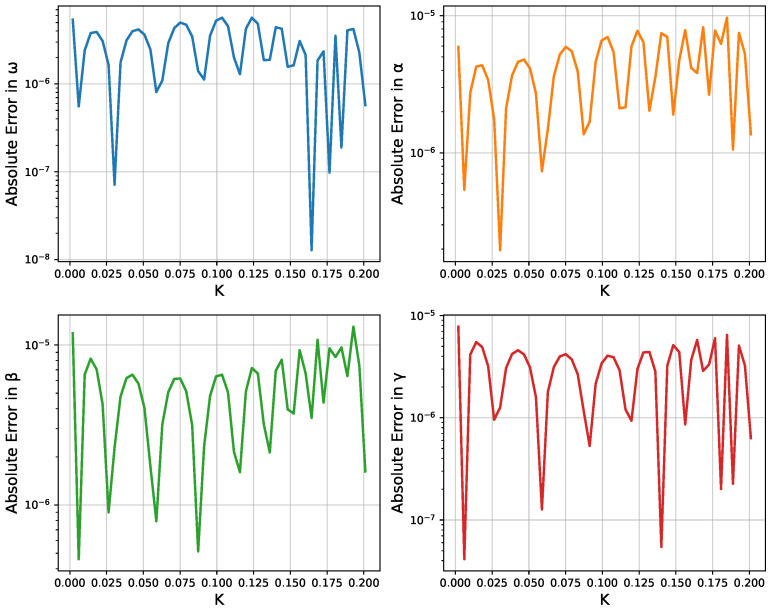
Log-scale plot of absolute errors between the neural network predictions and the individually optimized parameter values.

Finally, Figure 6 illustrates the absolute errors

$$\begin{array}{cc} \; & |\omega_{\mathrm{NN}}\left(K\right)-\omega_{\mathrm{ref}}\left(K\right)|,\;|\alpha_{\mathrm{NN}}\left(K\right)-\alpha_{\mathrm{ref}}\left(K\right)|,\\ \; & |\beta_{\mathrm{NN}}\left(K\right)-\beta_{\mathrm{ref}}\left(K\right)|,\;|\gamma_{\mathrm{NN}}\left(K\right)-\gamma_{\mathrm{ref}}\left(K\right)| \end{array}$$

across the sampled *K*-values, plotted in a log scale. These small errors confirm that the neural networks successfully learn the parameter dependencies over the entire range $\left(0,\tilde{K}\right]$.

### 3.5. Maximum Deflection Comparison with Exact Solution

In order to further assess the accuracy of the approximate Fourier solutions, we compared the maximum (peak) amplitude of each Fourier solution against the exact amplitude obtained from the transcendental equation (see Equation (16))

(21) $$-A^{2}-2K\ln\left(1-A\right)=0\;,$$

where we ignore the trivial solution A=0. For each sampled value of *K*, we used a standard root-finding method (Brent’s method) to solve for the nontrivial maximum deflection $A_{\mathrm{ref}}$. Next, we optimized the Fourier parameters θ=[ω,α,β,γ] with 2000 Adam steps, constructed the corresponding Fourier approximate solution and extracted its maximum deflection $A_{\mathrm{Fourier}}$. The procedure was repeated for 20 values of *K* in the range $\left(0,K^{*}\right)$.

**Figure 7:**
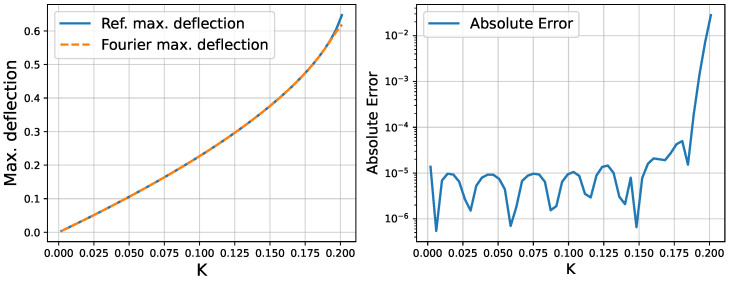
(**Left**) Comparison of the reference maximum deflection (solid line) derived from solving $-A^{2}-2K\ln\left(1-A\right)=0$ with the Fourier-based maximum deflection (dashed line). (**Right**) The absolute error between these two maximum deflections displayed on a logarithmic scale.

Figure 7 illustrates the results. The left panel shows both the true amplitude (solid line) and the Fourier-based amplitude (dashed line) as functions of *K*. The right panel plots the absolute difference $|A_{\mathrm{Fourier}}-A_{\mathrm{ref}}|$ in a logarithmic scale. We observe that the approximation remains accurate over the entire range of *K*, with the absolute error staying within a small margin for practical purposes.

Notice that the exact maximum deflection can be expressed in terms of Taylor expansion using Equation (21) and implicit differentation:

(22) $$A=2K+2K^{2}+\frac{14}{3}K^{3}+14K^{4}+\frac{2138}{45}K^{5}+\frac{1562}{9}K^{6}+\ldots \;.$$

This result was obtained in *Mathematica* [25] by employing AsymptoticSolve.

## 4. Extension to Non-Zero Geometric Parameter

The approach described in Section 3 focused on the limiting case ξ→0, where the governing equation simplifies to Equation (12). In practical magMEMS design, however, the geometric parameter ξ=b/L is typically non-zero and can significantly affect the system dynamics. This section extends our Fourier neural network method to handle the general case with arbitrary ξ>0, using the full governing Equation (4).

### 4.1. Generalized Fourier Approximation for Arbitrary ξ

For non-zero ξ, the governing Equation (4) can be rewritten as:

(23) $$\frac{\ddot{x}+x}{\tau (x,\xi )}=K\;,$$

where τ(x,ξ) is defined as:

(24) $$\tau \left(x,\xi \right)=\frac{\xi^{2}\left(1-x\right)}{\sqrt{\xi^{2}{\left(1-x\right)}^{2}+1}}+\frac{1}{\left(1-x\right)\sqrt{\xi^{2}{\left(1-x\right)}^{2}+1}}-\xi \;.$$

We employ the same Fourier ansatz as in Equation (19):

(25) $$\hat{x}\left(t\right)=\alpha \sin^{2}\left(\omega t\right)+\beta \sin^{2}\left(2\omega t\right)+\gamma \sin^{2}\left(3\omega t\right)\;,$$

which automatically satisfies the initial conditions $\hat{x}\left(0\right)=\dot{\hat{x}}\left(0\right)=0$. The key difference is that now we must optimize the parameter vector θ=[ω,α,β,γ] for each pair (K,ξ) rather than just *K* alone.

The residual function becomes:

(26) $$R_{\theta }\left(t;K,\xi \right)=\frac{\ddot{\hat{x}}\left(t\right)+\hat{x}\left(t\right)}{\tau (\hat{x}\left(t\right),\xi )}-K\;,$$

and the loss function is defined as:

(27) $$L\left(\theta ;K,\xi \right)=\frac{1}{N}\sum_{i=1}^{N}{\left[R_{\theta }\left(t_{i};K,\xi \right)\right]}^{2}\;.$$

### 4.2. Two-Input Neural Network Architecture

**Figure 8:**
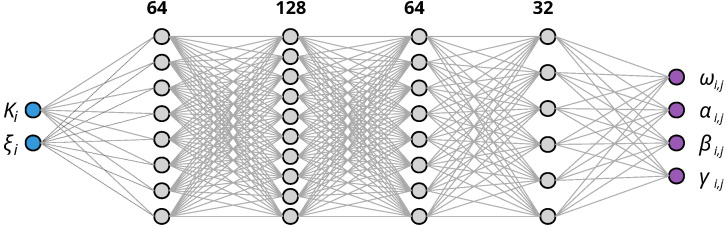
Neural network architecture for mapping $\left(K_{i},\xi_{j}\right)$ to Fourier parameters [ω,α,β,γ]. The network uses two inputs for the general case (ξ≠0) compared to one input (*K* only) when ξ=0.

To efficiently handle the extended parameter space, we train a neural network that maps from the two-dimensional input (K,ξ) directly to the four Fourier parameters [ω,α,β,γ] (see Figure 8). This contrasts with the one-dimensional input *K* used in the ξ→0 case. The same surrogate rationale from Stage 2 applies here: once trained, the map (K,ξ)↦[ω,α,β,γ] provides instantaneous, differentiable evaluations that amortize per-query optimization and enable gradient-based inverse design across the two-parameter space.

The neural network architecture consists of fully connected layers with widths and GELU activations. We optimize the parameters for a grid of (K,ξ) pairs where K∈[0.01,0.19] and ξ∈[0.1,2.0], ensuring that all combinations remain below the dynamic pull-in threshold $K_{\xi }^{*}$.

#### Workflow Overview and Grid Summary

The procedure follows two concise steps: (i) direct optimization of θ=[ω,α,β,γ] for each $\left(K_{i},\xi_{j}\right)$ on a coarse grid (no neural network in this step), producing a dataset (K,ξ)↦(ω,α,β,γ); and (ii) training a feedforward neural network to model this mapping. In the extended setting, the NN has two inputs (K,ξ) and four outputs (ω,α,β,γ), compared to one input (*K* only) when ξ=0.

We use 20 points in *K* and 20 points in ξ (i.e., 400 total pairs) (Table 1).

### 4.3. Validation Against High-Precision Solutions

**Figure 9:**
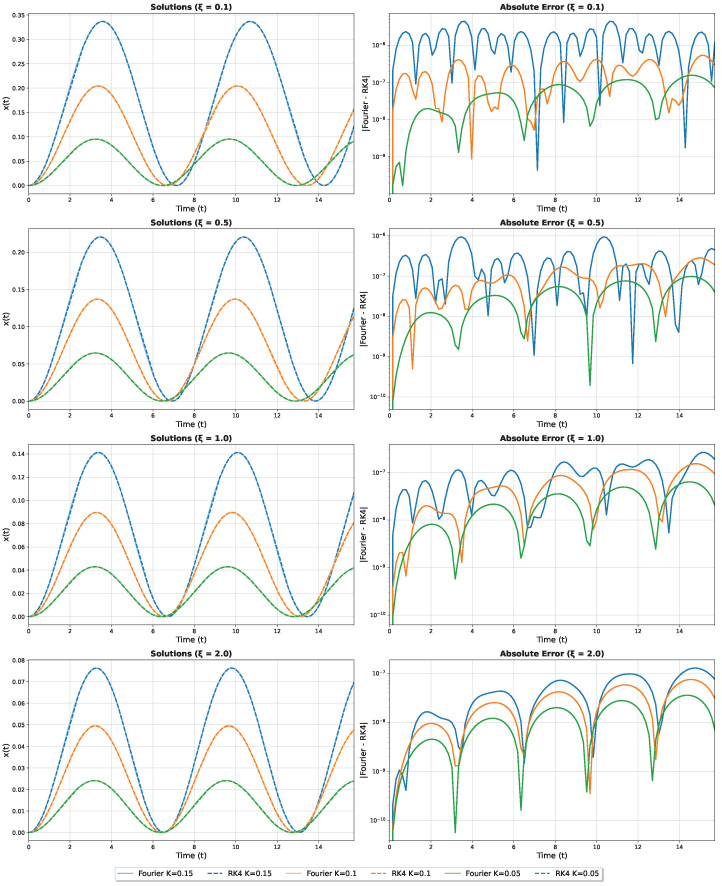
Validation of Fourier approximations for non-zero ξ values. Left panels: Comparison between Fourier solutions (solid lines) and RK4 solutions (dashed lines). Right panels: Absolute errors on logarithmic scale. Each row corresponds to a different ξ value: 0.1, 0.5, 1.0, and 2.0.

Figure 9 presents a comprehensive validation of our approach for different values of ξ. For each ξ∈{0.1,0.5,1.0,2.0} and various *K* values, we compare the Fourier approximation against high-precision Runge-Kutta solutions. The left panels show excellent agreement between the methods, while the right panels display the absolute errors on a logarithmic scale, demonstrating the high accuracy of our approach across different geometric configurations.

### 4.4. Parameter Dependency Analysis

**Figure 10:**
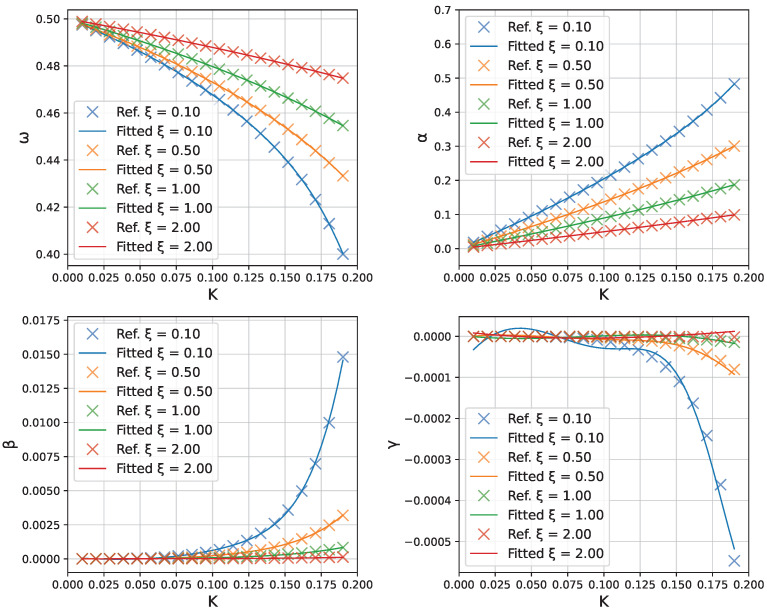
Fourier parameters as functions of *K* for different ξ values. Crosses: individual optimization results. Solid lines: neural network predictions. The geometric parameter ξ significantly influences the parameter trends, particularly for β and γ.

The trained neural network enables efficient exploration of how the Fourier parameters vary with both *K* and ξ. Figure 10 illustrates the dependence of each parameter [ω,α,β,γ] on *K* for selected values of ξ. The crosses represent the reference values obtained from individual optimizations, while the solid lines show the neural network predictions.

The results reveal that the geometric parameter ξ has a pronounced effect on the parameter dependencies. In particular, the higher-order harmonic coefficients β and γ show significant variation with ξ, indicating that the geometric ratio between conductor separation and length plays a crucial role in determining the oscillatory behavior.

The fitted trends are consistent with the magnetostatic softening process. The fundamental frequency ω decreases almost linearly with *K*, with a steeper slope for smaller ξ. The leading amplitude α grows roughly linearly with *K* and is suppressed as ξ increases, reflecting a weaker effective forcing. Higher harmonics remain small, that is, β increases with *K* and is most pronounced for small ξ, whereas γ stays close to zero and vanishes as ξ grows. Consequently, for larger ξ the response is nearly single-harmonic, which explains the improved accuracy observed in Figure 9.

**Figure 11:**
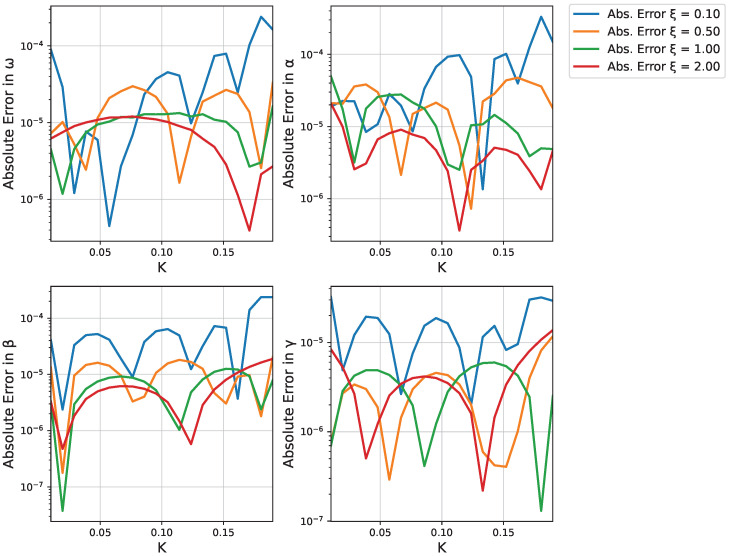
Absolute errors between neural network predictions and reference parameter values for different ξ values, displayed on logarithmic scale. The errors remain small across all parameters and ξ values.

Figure 11 quantifies the accuracy of the neural network predictions by showing the absolute errors between predicted and reference values. The errors remain small across the entire parameter space, confirming the reliability of our approach.

### 4.5. Three-Dimensional Parameter Surfaces

**Figure 12:**
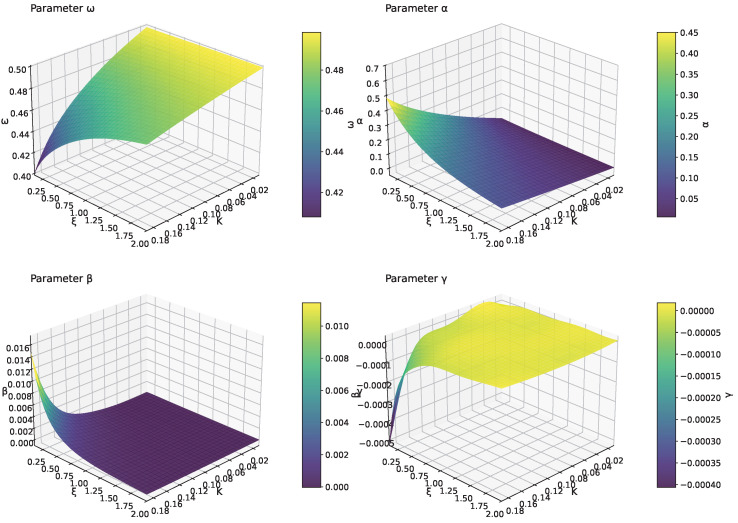
Three-dimensional parameter surfaces showing the variation of Fourier parameters [ω,α,β,γ] with both excitation parameter *K* and geometric parameter ξ. These surfaces provide comprehensive insight into the parameter dependencies for magMEMS design.

To provide a comprehensive view of the parameter space, Figure 12 presents three-dimensional surface plots showing how each Fourier parameter varies simultaneously with both *K* and ξ. These surfaces capture the full parameter dependencies and can serve as visual guides for magMEMS design optimization.

The surface plots show that the frequency parameter ω increases smoothly with both *K* and ξ, exhibiting a nearly linear relationship across the parameter space. The primary amplitude coefficient α displays a more complex behavior, decreasing with increasing ξ while showing nonlinear dependence on *K*. The higher-order harmonic coefficients β and γ remain relatively small, with β showing the most variation across the parameter space and γ remaining close to zero throughout most of the domain. All parameters demonstrate smooth, well-behaved surfaces without discontinuities, ensuring the numerical stability of the approach.

This extended framework provides magMEMS designers with a comprehensive tool for analyzing devices with arbitrary conductor geometries, enabling rapid optimization across the full design space defined by both electrical and geometric parameters.

Alternatives such as low-order polynomials, splines, radial-basis interpolation, or Kriging/Gaussian process (GP) can approximate smooth maps very well, but in our two-parameter input (K,ξ) we observed: (i) nonuniform sensitivity and mild nonlinearity near the pull-in boundary; (ii) cross-coupling between *K* and ξ; and (iii) the need for stable analytic gradients. A small fully connected NN handled these without hand-crafted basis selection, provided smooth, differentiable predictions, and generalized well between grid points. Interpolation-based surrogates typically require denser, more regular grids and may introduce non-smooth derivatives at knots, while GP/Kriging adds kernel selection and $O(n^{3})$ scaling. The compact NN achieved comparable or better accuracy with straightforward training and O(1) inference.

## 5. Proof-of-Concept Measurement and Error Budget

### 5.1. Prototype Experimental Set-Up

**Figure 13:**
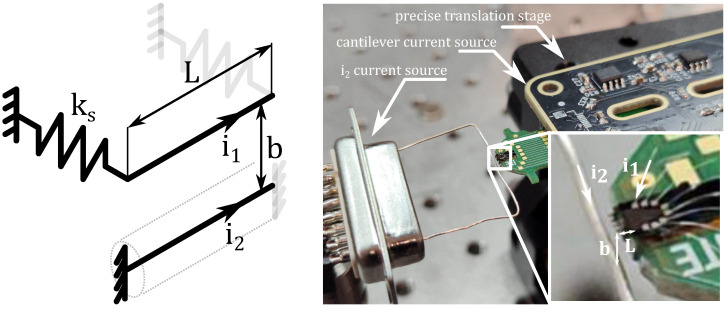
Simplified representation and photograph of the experimental setup with an elastic microcantilever carrying a current-leading filament.

The coil wire was driven with a DC current of 4A, while the magMEMS cantilever carried a DC bias on the order of milliamperes (Figure 13). Displacement was recorded using a SIOS^TM^ Nano Vibration Analyzer (Ilmenau, Germany), a laser Doppler vibrometer with a noise floor of $5\;\mathrm{pm}/\sqrt{\mathrm{Hz}}$. Since the instrument’s DC path is suppressed, purely static deflection could not be measured directly. Instead, the quasi-DC response was inferred under sub-resonant harmonic actuation. The calibrated quasi-static sensitivity is $S_{\mathrm{stat}}=\frac{\mu_{0}\mu_{r}i_{1}L}{2\pi bk_{s}}\approx 6.46\times 10^{-2}\;\mathrm{nm}/\mathrm{mA}$(≈65pm/mA).

In the present large-gap regime, the nondimensional groups obtained from Table 2 satisfy $K≃4.01\times 10^{-7}$ and $\xi ≃3.10\times 10^{2}$, from which it follows that $R^{\prime }\left(x_{0}\right)\leq K/\xi \approx 1.3\times 10^{-9}\ll 1$. The linearization of (4) about the DC equilibrium $x_{0}$ yields

$$\delta \ddot{x}+\left(1-R^{\prime }\left(x_{0}\right)\right)\;\delta x=f\left(t\right),\;R^{\prime }\left(x\right)=\frac{K}{{\left(1-x\right)}^{2}\sqrt{\xi^{2}{\left(1-x\right)}^{2}+1}},$$

so $k_{\mathrm{eff}}=k_{s}\left(1-R^{\prime }\left(x_{0}\right)\right)\approx k_{s}$. Consequently, the quasi-static gain is independent of the DC bias:

$$X_{\mathrm{rms}}=\frac{1}{\sqrt{2}}\frac{\mu_{0}\mu_{r}\;i_{1}\;i_{2}^{\mathrm{ac}}\;L_{\mathrm{eff}}}{2\pi \;b\;k_{s}}\;\frac{1}{1-R^{\prime }\left(x_{0}\right)}\approx \frac{1}{\sqrt{2}}\frac{\mu_{0}\mu_{r}\;i_{1}\;i_{2}^{\mathrm{ac}}\;L_{\mathrm{eff}}}{2\pi \;b\;k_{s}}.$$

Thus any observable DC trend is attributed to a weak DC dependence of parasitic AC ripple and the readout chain rather than elastic/magnetic softening.

Measured RMS contains signal and a DC-independent baseline. Using the $i_{2}\;=\;0$ mA point as baseline, $X_{\mathrm{noise}}=5.54$ pm, the signal-only RMS is computed in the power domain as

$$X_{\mathrm{sig}}=\sqrt{\max\;\left(X_{\mathrm{meas}}^{2}-X_{\mathrm{noise}}^{2},\;0\right)}.$$

With the quasi-static gain $G=S_{\mathrm{stat}}/\sqrt{2}\approx 46\;\mathrm{pm}/\mathrm{mA}$, the equivalent ripple current is $i_{2,\mathrm{ripple}}=X_{\mathrm{sig}}/G$.

The experimental parameters were extracted from Table 2, and the excitation parameter *K* was computed via Equation (5). For completeness, the nondimensional groups read $\xi =b/L\approx 3.10\times 10^{2}$ and $K=\mu_{0}\mu_{r}i_{1}i_{2}L/\left(2\pi k_{s}b^{2}\right)\propto i_{2},$ so $K\approx 4.01\times 10^{-7}$ at $i_{2}=10\;\mathrm{mA}$. Since $R^{\prime }\left(0\right)\approx K/\xi ∼10^{-9}$, the operation is overwhelmingly far from pull-in, and the quasi-static RMS is flat versus DC bias to first order. Thus, any residual variation reflects unintended AC ripple or environmental excitation.

We then analyzed the vibration spectrum. With a DC current applied to the magMEMS cantilever, the first-harmonic RMS amplitude varied by at least a factor of five, from 1.6pm under repulsive bias to 7pm under attractive bias; see Table 3. For clarity, Table 3 reports the measured RMS values together with the estimated uncertainty Δ.

This example illustrates a limitation of the Fourier approach at very small excitation: for the present range of the excitation parameter, the relative error of higher Fourier coefficients deteriorates the approximation. Nevertheless, one can compute the maximum deflection numerically as the root of $f_{K,\xi }\left(s\right)$ in Equation (8). For the case considered we obtain $s=6.476\;\cdot \;10^{-10}$, which yields $1.043\;\cdot \;10^{-12}\;m\;$} as the maximum deflection.

### 5.2. Experimental Perspective

**Figure 14:**
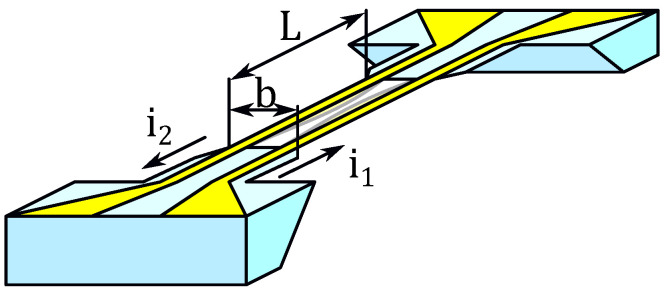
Idea of a MagMEMS—model device, with mechanical and electric structure simplified to the basic case—two conductors.

To validate the findings obtained with the test Fourier neural network, a model magMEMS device is required. The simplest configuration, both in form and function, consists of two parallel conductor wires positioned opposite one another. These wires are self-supporting, serving simultaneously as conductors and as mechanical scaffolding. To regulate their length and stiffness, the conductors are fabricated in the form of ribbons and placed on an insulating substrate, with certain sections extending partially across a gap. Furthermore, the wires may be curved or bent, enabling them to contract or expand in response to external stimuli. A schematic illustration of the structure is presented in Figure 14.

The primary advantage of the proposed structure lies in its relative simplicity of fabrication and operation. Detection of the desired wire deflection induced by the self-generated magnetic field must be carried out using a high-resolution microscopic technique such as scanning electron microscopy (SEM), for example.

**Figure 15:**
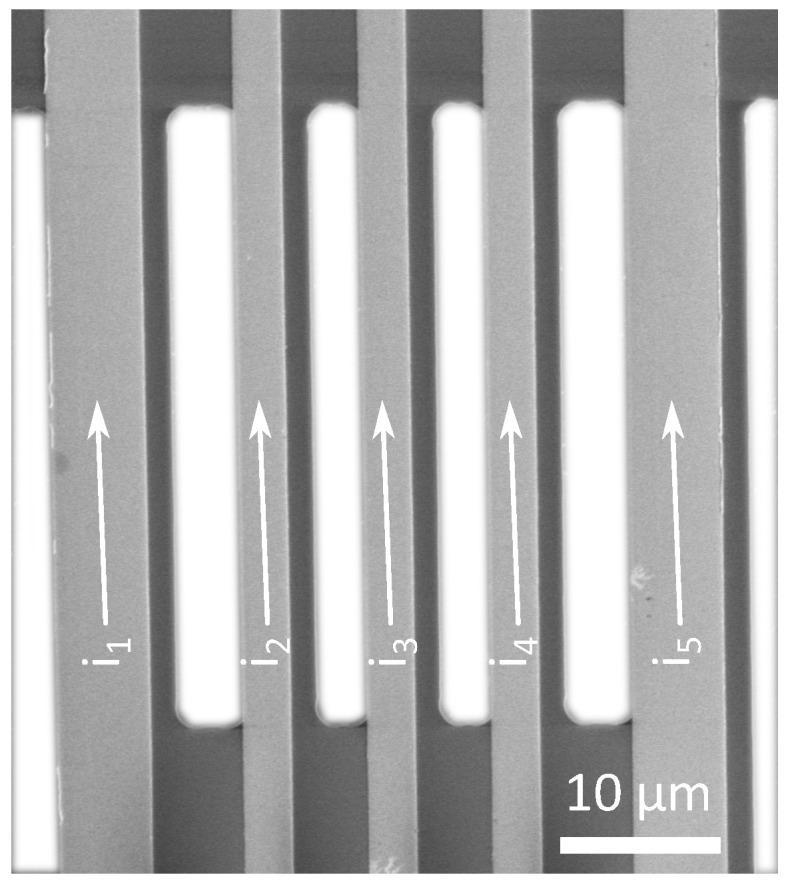
SEM image of an realized, but unoptimized exemplary structure for in-principle observation and validation of the calculated parameters.

The initial test structures were fabricated with L=40μm and b=5μm, as illustrated in Figure 15. To ensure mechanical stability and to prevent wedging, the structure may be produced with more conductive lines than strictly required; however, in the verification experiment, only two lines were employed. The stiffness of the structure is governed by the line thickness, which in this case was set to 3μm, yielding a stiffness of approximately 10N/m. Based on the general model, the design parameters can subsequently be optimized to satisfy the Fourier parameters defined by the three-term ansatz in Equation (19).

## 6. Conclusions

Approximate oscillatory solutions for a magMEMS switch with current-carrying conductors are obtained using the Fourier Neural Network Method for both the limiting case $\xi \to 0^{+}$ and the general case with arbitrary geometric parameter ξ>0. Approximate periodic solutions for the range of the excitation parameter values less than the pull-in value exhibit accuracy comparable to that of classical ODE solvers. Numerical illustrations and validations of the effect of both the scaled excitation parameter *K* and geometric parameter ξ on the oscillation characteristics were presented. This allows for rapid evaluation of magMEMS switch device performance for various excitation currents, filament lengths, maximum separation distances, and spring parameters. The extension to non-zero ξ significantly broadens the applicability of the method, enabling comprehensive design space exploration. These results are especially useful in system optimization and can be easily implemented in a time domain circuit simulator like Spice.

MagMEMS devices under consideration are uniform in material composition, which is not the universal case. Inclusion of auxiliary materials (e.g., support, insulating, forming, etc.) might prove model less applicable by shear change to wire-wire *b* dimension. However, the most ubiquitous form of RF switches, nanowire-based circuits, or active MEMS devices are formed of a single material only [8]. As such, they are most accurately described by the introduced model. We believe that the nanofield is one of the main application areas.

The results obtained in this work can be useful for the design of magMEMS based on current-carrying filaments. Applications include design of magMEMS switches, oscillators, and resonators. The next research phase will involve a comprehensive experimental study of oscillating current-carrying filaments, transitioning from scaled-up prototypes to application-ready magMEMS devices. To develop a more complete model, future studies should explore damping and nonlinear resonances in harmonically excited systems.

## Figures and Tables

**Table 1:** Illustrative 20×20 grid of (K,ξ) pairs. Each entry stores the optimized parameter vector $\theta_{i,j}=\left[\omega ,\alpha ,\beta ,\gamma \right]$ obtained in Step 1 of optimization algorithm.

	$K_{1}$	$K_{2}$	*…*	$K_{20}$
$\xi_{1}$	$\theta_{1,1}$	$\theta_{1,2}$	*…*	$\theta_{1,20}$
$\xi_{2}$	$\theta_{2,1}$	$\theta_{2,2}$	*…*	$\theta_{2,20}$
⋮	⋮	⋮	⋱	⋮
$\xi_{20}$	$\theta_{20,1}$	$\theta_{20,2}$	*…*	$\theta_{20,20}$

**Table 2:** Variables and parameters, updated with the correct gap and active length.

Variables and Parameters	Value	Units	Description
$i_{1}$	4	[A]	current in coil wire
$i_{2}$	$10\times 10^{-3}$	[A]	current in cantilever, used for *K* below
*b*	$1.61\times 10^{-3}$	[m]	effective coil–cantilever separation
*L*	$5.20\times 10^{-6}$	[m]	effective active length
$k_{s}$	0.04	[N/m]	spring constant of cantilever
$\mu_{0}$	$4\pi \times 10^{-7}$	$\left[N/A^{2}\right]$	vacuum permeability
$\mu_{r}$	1	[−]	relative permeability
$K=\frac{\mu_{0}\mu_{r}i_{1}i_{2}L}{2\pi \;k_{s}\;b^{2}}$	$4.01\times 10^{-7}$	[−]	excitation at $i_{2}=10$ mA
$\xi =\frac{b}{L}$	$3.096\times 10^{2}$	[−]	geometric parameter
$S_{\mathrm{stat}}=\frac{\mu_{0}\mu_{r}\;i_{1}\;L}{2\pi \;b\;k_{s}}$	$6.46\times 10^{-2}$	[nm/mA]	quasi-static sensitivity (≈65pm/mA)

**Table 3:** RMS vibration under DC bias with power-domain baseline removal and implied equivalent ripple.

$i_{2}$ DC (mA)	$X_{\mathrm{meas}}$ (pm)	±Δ (pm)	$X_{\mathrm{sig}}$ (pm)	$i_{2,\mathrm{ripple}}$ (mA)
0	5.54	1.11	0.00	0.000
2	4.29	0.86	0.00	0.000
4	6.95	1.39	4.20	0.092
6	8.46	1.69	6.40	0.140
8	6.74	1.35	3.80	0.083
10	7.19	1.44	4.60	0.100

Baseline $X_{\mathrm{noise}}=X_{\mathrm{meas}}\left(0\;\mathrm{mA}\right)=5.54\;\mathrm{pm}$. Signal-only $X_{\mathrm{sig}}=\sqrt{\max(X_{\mathrm{meas}}^{2}-X_{\mathrm{noise}}^{2},\;0)}$. Gain $G=S_{\mathrm{stat}}/\sqrt{2}\approx 46\;\mathrm{pm}/\mathrm{mA}$. Equivalent ripple $i_{2,\mathrm{ripple}}=X_{\mathrm{sig}}/G$.

## Data Availability

The code for this project is available at: https://github.com/armanbolatov/magmems_optimization (accessed on 22 November 2025). The data supporting the reported results can be reproduced using the provided code.
